# Exposure to airborne SARS-CoV-2 in four hospital wards and ICUs of Cyprus. A detailed study accounting for day-to-day operations and aerosol generating procedures

**DOI:** 10.1016/j.heliyon.2023.e13669

**Published:** 2023-02-11

**Authors:** Rafail Konatzii, Fabian Schmidt-Ott, Lakis Palazis, Panagiotis Stagianos, Maria Foka, Jan Richter, Christina Christodoulou, Jean Sciare, Michael Pikridas

**Affiliations:** a Climate and Atmosphere Research Centre, The Cyprus Institute, Nicosia, 2121, Cyprus; b Department of Intensive Care Unit, Nicosia General Hospital, Ministry of Health, Nicosia, 2029, Cyprus; c Department of Molecular Virology, The Cyprus Institute of Neurology and Genetics, Nicosia, 1683, Cyprus; d Department of Physics, University of Helsinki, Helsinki, 00014, Finland

**Keywords:** Airborne SARS-CoV-2 fast detection, Occupational health, Aerosol generating procedures, SARS-CoV-2 size distribution

## Abstract

In any infectious disease, understanding the modes of transmission is key to selecting effective public health measures. In the case of COVID-19 spread, the strictness of the imposed measures outlined the lack of understanding on how SARS-CoV-2 transmits, particularly via airborne pathways. With the aim to characterize the transmission dynamics of airborne SARS-CoV-2, 165 and 62 air and environmental samples, respectively, were collected in four COVID-19 wards and ICUs in Cyprus and analyzed by RT-PCR. An alternative method for SARS-CoV-2 detection in air that provides comparable results but is less cumbersome and time demanding, is also proposed. Considering that all clinics employed 14 regenerations per hour of full fresh air inside patient rooms, it was hypothesized that the viral levels and the frequency of positive samples would be minimum outside of the rooms. However, it is shown that leaving the door opened in patient rooms hinders the efficiency of the ventilation system applied, allowing the virus to escape. As a result, the highest observed viral levels (135 copies m^−3^) were observed in the corridor of a ward and the frequency of positive samples in the same area was comparable to that inside a two-bed cohort. SARS-CoV-2 in that corridor was found primarily to lie in the coarse mode, at sizes between 1.8 and 10 μm. Similar to previous studies, the frequency of positive samples and viral levels were the lowest inside intensive care units. However, if a patient with sufficient viral load (Ct-value 31) underwent aerosol generating procedures, positive samples with viral levels below 45 copies m^−3^ were acquired within a 2 m distance of the patient. Our results suggest that a robust ventilation system can prevent unnecessary exposure to SARS-CoV-2 but with limitations related to foot traffic or the operations taking place at the time.

## Highlights

• Foot traffic in hospital wards hinders the ability of the ventilation system to act as a protective measure.
• Airborne SARS-CoV-2 particles inside clinics identified at sizes 1–2.5 μm, which allow them to travel distances longer than a few meters.
• AGP are a source of SARS-CoV-2 spread, which is non-uniform in space.
• Rapid detection method of airborne SARS-CoV-2, which can act as an early warning system, is presented.

## 1 Introduction

The most significant pandemics of the 20th century took place in 1918, 1957, and 1968 and were caused by influenza viruses. In contrast, the 21st century has been marked by alerts concerning atypical pneumonias caused by corona viruses. In 2003, a global alert was issued for–a yet unknown—illness caused by a novel coronavirus [1] that became known as severe acute respiratory syndrome (SARS), resulting in approximately 800 casualties with a mortality rate of 10%. In 2012, a similar disease named Middle East respiratory syndrome occurred in Saudi Arabia with a mortality rate of 35%. On March 11, 2020, the World Health Organization (WHO) declared the ongoing spread of the Severe Acute Respiratory Syndrome Coronavirus 2 (SARS-CoV-2) a global pandemic, whose death toll has caused at least 55 countries to order a lockdown. Unlike previous SARS inducing viruses, SARS-CoV-2 can be effectively transmitted airborne even by presymptomatic patients [2], a feature that boosted the spread of the disease to become a worldwide epidemic [3].

On the frontline of this epidemic are healthcare professionals who have been tasked to treat those infected. Due to the elevated risk of occupational exposure, precautionary measures have been recommended at healthcare units. These include treating COVID-19 patients in negative pressure rooms where the air is exchanged at least six times per hour if a nominal of 15 air exchanges per hour cannot be achieved [4]. The use of surgical masks by COVID-19 patients additionally reduces the chance of transmission in most cases [5,6], especially when other precautionary measures cannot be available. Early on during the pandemic, nosocomial transmission of SARS-CoV-2 was reported [7], and since similar episodes have been published [[8], [9], [10], [11], [12]], but defining the causes of transmission remains a challenge [13,14]. At hospitals in China, an infection rate ranging between 2 and 29% has been reported [7,15,16]. In the UK, healthcare workers exhibited elevated seroconversion rates compared to the general population suggesting that they were either infected or, as a minimum, exposed to SARS-CoV-2 [17,18].

In any infectious disease, understanding the modes of transmission is a key factor in protecting healthcare workers and implementing effective public health measures [14,19]. The lack of understanding of the SARS-CoV-2 transmission dynamics was a major factor that led to the imposed isolation precautions recommended by public health authorities worldwide. What makes SARS-CoV-2 stand out is its feature to spread from presymptomatic and asymptomatic patients [2], via aerosol in addition to direct contact and cough droplets. If the surrounding environment is dry enough, which is the typical case, cough droplets will, reduce in size [20] prolonging their lifetime [21]. However, viral aerosol can be directly produced in the absence of cough [22] by individuals who are COVID-19 positive when they breath, speak [[23], [24], [25], [26]] or sing [27,28].

The type of expiratory activity determines, to a large extent, the number and size distribution of the produced aerosol, with smaller sizes being associated with deeper parts of the respiratory tract [19]. The actual size of SARS-CoV-2 is approximately 120 nm in diameter [4] but because it is emitted as a humid particle its size in the air is larger and still under investigation. There is no consensus at which sizes the virus is found. The first report on SARS-CoV-2 size distribution suggested that the virus lies mainly in the submicron range [29] followed by another study placing it in supermicron sizes only [30]. Santarpia et al. [22] identified positive samples in the entire size spectrum, including sizes below 1 μm. A more recent study [31] suggested that the size range associated with the virus, depended on hospital ward (or the conditions applied in each ward), such that viral content was identified in the coarse mode (between 2.5 and 10 μm) in normal care units but above 10 μm inside ICUs. Unlike the aforementioned publications, the majority of nosocomial studies relied on total suspended particulate (TSP) air samples without any information on size [[32], [33], [34], [35], [36], [37]]. In several cases the virus was not detected at all [[38], [39], [40], [41], [42], [43]]. Low to zero viral levels in negative pressure rooms inside ICU has been reported [[44], [45], [46], [47]], suggesting that precautionary measures, even though difficult to implement, are effective and curb viral levels in the air. Despite the applied measures, clinicians have been raising concerns about how aerosol generating procedures (AGPs), a family of procedures in critically ill patients can promote the generation of aerosol, contribute to the viral load. When applied, AGPs are supposed to cause episodic elevation of the aerosol population surrounding the patient while clinicians are in proximity, exposing them to direct hazard. It should be noted that it is still debated among healthcare workers which treatments fall under AGP, even though the WHO provides a categorization [48]. Very few studies have targeted AGPs on COVID-19 patients [12,37,49,50], each of which studied a different procedure.

This work is a multi-goal study aiming to characterize airborne SARS-CoV-2 inside hospital clinics. For this purpose, more than 165 air samples and 62 environmental samples have been analyzed in three different wards of two hospitals in Cyprus. The primary goal was to characterize the occupational hazards clinicians faced at Normal Care Unit (NCU) and Intensive Care Unit (ICU). We report on the ventilation characteristics, which is an essential protective measure, of the sampled locations, that has been neglected in most studies so far, and demonstrate how its ability to protect clinicians can be hindered. A complete characterization requires information on the size fraction the airborne virus can be found at, which sets the second goal, discussed in Section 4.2. Since COVID-19 is a pulmonary disease whose patients are subjected to AGP, care was taken that a few samples targeted such procedures setting the third and most tedious goal (Section 4.4). Like most studies, filter-based methods were employed but with a simple alteration to the traditional sampling technique (Section 2.1) to minimize the problem of structural damage during sampling. To the best of our knowledge, the majority of SARS-CoV-2 related studies relied on RT-PCR as the analytical technique. Even though this study does not go against the norm, it also proposes an alternative method, described in Section 2.3, which in combination with a cyclonic sampler can serve as a warning system on airborne SARS-CoV-2.

## 2 Methods

### 2.1 Samplers

A suite of six different sampling techniques, summarized in Table 1, was employed in this study, with the aim to collect both air and environmental samples. When sampling took place inside patient rooms, all air samplers were located at an elevation of 1–1.5 m above ground and no more than 0.5 m away from at least one patient.

**Table 1:** Technical characteristics of air and environmental samplers employed in this study.

	**Flow (L m**^**−1**^**)**	**Collection Substrate**	**Sample duration**	**D**_**50**_**(nm)**
**Filter sampler**	5–6	Teflon or Gelatine filters	1 h–24 h	Total Suspended Particles
**Coriolis Compact**	50	polypropylene cone	8 h	500
**Coriolis Micro**	150	polypropylene cone	1 h	500
**MOUDI**^a^**Impactor**	28	Teflon filters	8 h–72 h with intervals	320^b^
**Surface samples**	–	Dacron swabs	–	–
**Passive samples**	–	Petri dishes	4–10 days	–

a Microorifice Uniform Deposit Impactor

b This is the lowest size cut of this impactor.

Filter sampler: the majority of air samples were attained using a custom-made atmospheric sampler that employed a pump (KNF Model N86KT.18) connected to a polypropylene filter holder (Advantec 43303020) using PTFE (FHLP04700) or gelatin (SKC 225–9552) substrates. The former substrates were used if sampling duration exceeded 1 h while the latter were utilized if the duration was equal to 1 h or less. The sampling flow ranged from 5.6 to 6.1 L min^−1^, (median sample flow equal to approximately 5.8 L min^−1^) depending on the unit employed. The sample flow stability was ensured by checking the flow prior and after sampling on-site using a mass flow meter (TSI Model 4143F). In case these two readings differed by 0.5 L min^−1^ or more the sample was rejected. In this study, the filter holders employed were modified to operate as open face with the entire surface of the substrate being exposed directly to the sampled air (Suppl. Fig. 1). This modification reduced the impact velocity of collected particles without compromising the flow, while reducing noise levels of the sampling system.

The Coriolis Compact sampler: is a cyclonic sampler by Bertin Instruments that collects particles with aerodynamic diameters greater than 500 nm (D_50_ at 500 nm) in a reusable cone that serves as a cyclone. It is operated at 50 L min^−1^ at dry conditions (no liquid is added to the cone) and will thus be referred to as the dry cyclonic sampler.

The Coriolis Micro sampler: is another cyclonic sampler by Bertin Instrument which is operated at wet conditions at a flow rate of 150 L min^−1^. A volume of 10 mL phosphate buffer was added to the cone prior to sampling, serving as the collection media for the SARS-CoV-2 virus. After a sampling of 1 h, approximately 1 mL of particle phosphate solution remained, enriched by particles taken up from the air. It is assumed that the efficacy of collection and extraction were higher compared to the samples collected using a Coriolis Compact, where no liquid media was used. The Coriolis Micro samplers will be referred to as the wet cyclonic sampler.

The microorifice uniform deposit impactor [51] (MOUDI): was used to collect samples in 7 size segregated stages (TSI Model 100NR) with a D_50_ (particles collected with 50% efficiency) at 10, 5.6, 3.2, 1.8, 1.0, 0.56 and 0.32 under a 28 L min^−1^ flow. Typical MOUDI configurations include an eighth stage centered at 0.18 μm that was removed to reduce noise levels. Sampling was conducted for 6–8 h outside a four-patient room and only during morning hours when the patient room door was left open for a prolonged duration or opened frequently.

Environmental samples consisted of surface swab samples and passive samples. The surface samples were collected by swabbing approximately 25 cm^2^ areas of eachitem using Dacron swabs (Millipore MMSB10025) soaked in phosphate buffer. High-touch surfaces were selected for sampling, including bed rails, toilet seats, bins and door handles. Swabs were then transferred to 5 mL of phosphate buffer. The passive samplers were collected by positioning empty Petri dishes on the floor for collection of suspended particles deposited by gravitation. These were stationed at several locations inside FW, including the toilet of a 4-patient room, inside a 2-patient room in proximity to a patient, the apparel changing room and the corridor that connects these areas. Each Petri dish remained for a period of 4–10 days.

Filter holders, Petri dishes and reusable cones of cyclonic samplers were sterilized using absolute ethanol for analysis (Merck MFCD00003568) and left under UV light for 30 min inside a laminar flow hood equipped with a high-efficiency particulate air (HEPA) filter prior to sampling.

### 2.2 Sites

**Fig. 1:**
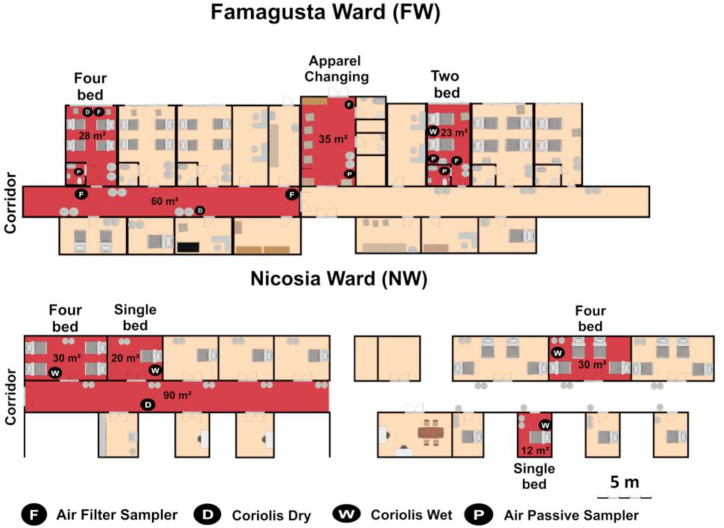
Schematic of Famagusta Ward (top) and Nicosia Ward (bottom) COVID-19 wards. Rooms where sampling took place are shown in red. The position of the samplers is also shown. The height of both clinics is 3 m.

Sampling was conducted inside wards of the Famagusta (35.062721 N, 33.972113E) and Nicosia (35.127766 N, 33.377286E) General Hospitals, both located in Cyprus, from November 19, 2020 until April 1, 2021. During the beginning of the sampling period, all symptomatic patients that required hospitalization were treated at the COVID-19 ward of Famagusta General Hospital, which will be referred to as Famagusta Ward (FW). The FW consisted of four four-bed, three two-bed and two single-bed rooms, one apparel changing area and three offices connected via a single corridor. A detailed floor plan of the rooms is shown in Fig. 1, including locations where samplers were collected. All patient rooms were equipped with an exhaust system operating continuously to achieve negative pressure conditions. However, as COVID-19 cases surged, additional COVID-19 wards were established in different hospitals in Cyprus, including one at the Nicosia General Hospital, which will be referred to as Nicosia Ward (NW), where sampling was also conducted. At NW, a ventilation system to achieve negative pressure conditions was employed, similar to the one at FW, in four and two-bed patient rooms.

The Nicosia General Hospital hosted all COVID-19 patients that required intensive care, initially at a 10-bed room of 214 m^2^, replaced by a 24-bed cohort of 327 m^2^, which will be referred as NIC1 and NIC2, respectively. Both ICUs were equipped with a ventilation system that allowed 16 air changes per hour using full fresh air. Patients inside either of the ICUs were intubated and subjected daily to aerosol generating procedures, unlike patients residing in FW and NW that were typically not intubated. A summary of the hospitals’ wards where sampling took place in this study, including the abbreviations used, can be found in Table 2. In each location, two field blank samples, at the beginning and end of the sampling period, were collected.

**Table 2:** Number of samples collected at each location excluding AGP samples. The number in parenthesis corresponds to the number of samples where SARS-CoV-2 RNA was identified.

				Air Samples			Environmental Samples	
**Hospital**	**Clinic Type**	**Abbreviation**	**Room type**	**Filter**	**Cyclonic**	**Cascade Impactor**	**Passive**	**Swabs**
Nicosia	Intensive	NIC1	Patient (214 m^2^)	8 (0)	N/A	N/A	N/A	N/A
General Hospital	Care	NIC2	Patient (327 m^2^)	17 (3)	N/A	N/A	2 (1)	N/A
								
Nicosia	Ward	NW	Patient	N/A	25 (6)	N/A	N/A	N/A
General Hospital		NW	Corridor	N/A	8 (1)	N/A	N/A	N/A
								
	Ward	FW	Patient 4-bed	24 (12)	8 (4)	N/A	6 (1)	8 (3)
		FW	Patient 2-bed	17 (5)	N/A	N/A	7 (3)	6 (0)
Famagusta Reference Hospital		FW	Apparel Changing	12 (4)	N/A	N/A	3 (0)	8 (1)
		FW	Corridor	29 (7)	9 (3)	8 (6)	5 (2)	17 (1)
								
		**Total**		**107(31)**	**50(14)**	**8(6)**	**23(7)**	**39(5)**

New admissions in either of the referred wards were subjected to RT-PCR test. While test results were pending, the patient was being isolated in a single room inside the ward, in which he/she remained for up to two days. Sampling was only conducted in proximity to adult patients with positive RT-PCR test and not to transient patients described above. Considering that SARS-CoV-2, infectiousness declines quickly within 7 days and initiates at least 2 days before symptom onset [52], this additional transient period of maximum two days resulted in sampling in proximity to patients who were confirmed symptomatic of SARS-CoV-2 during their declining infectiousness phase. This implies that the positivity frequency and viral load levels in this study are biased low. However, even though biased, they are representative of the daily routine of clinicians in wards and intensive care units, which was one of the aims of this study.

### 2.3 Analytical methods

RNA extraction and reverse transcription quantitative polymerase chain reaction (RT-qPCR) were performed following the CDC assay targeting the N-gene after extraction from the substrate, which could be filter, swab, passive collection media, or cyclonic centrifuge cones. For detection of SARS-CoV-2 RNA and the parallel detection of human DNA, an in-house duplex Real-Time RT-PCR assay was employed. Liquid samples obtained by the wet cyclonic sampler, were analyzed at the Nicosia General Hospital following a simpler protocol since no extraction was necessary. Detection was performed in one-step real-time format using the VIASURE SARS-CoV-2 Real-Time PCR Detection Kit. A detailed description of both analytical methodologies can be found in the supplement.

The concentration of each sample was calculated based on Eq. (1)

(1) $$C=r\frac{e_{v}}{s_{v}v_{air}}$$

where C is the concentration of the viral RNA in air (copies m^−3^), r is the viral RNA copies estimated by PCR, e_v_ (50 μL) and s_v_ (20 μL) are the volumes of the concentrated (extracted) sample and the volume of which was analyzed, respectively. The volume of air collected by each sample is denoted by v_air_ (m^3^).

Wet cyclonic samples were also analyzed by means of a Bio Electric Diagnostics (B.EL.D.) device [53], which is based on the cell-based biosensor technology known as the Bioelectric Recognition Assay [54] (BERA). The system uses analog to digital converters that monitor electrical variations caused by cells acting as bio-identification elements. B.EL.D. incorporates eight disposable carbon-screen printed electrodes (iMiCROQ SL, Tarragona, Spain or Metrohm, Herisau, Switzerland) where real-time changes in the electrical properties of these cells against a reference Ag/AgCl electrode are measured. The collected sample was divided to 20 μL, added to each of the 8 carbon electrodes, yielding 160 μL, the minimum volume required for the analysis. At each electrode, an additional 20 μL, each of which approximately accounting for 5 × 10^4^ of Vero membrane cells (LGC Promochem, ATCC CCL-81) made with monoclonal antibodies was added. Cell response was recorded as a time series of potentiometric measurements (in Volts) for 180 s at a sampling rate of 2 Hz. The 360 measurements produced are analyzed by a proprietary algorithm (Embio Diagnostics) that yielded Boolean results on the presence of SARS-CoV-2. The connection of B.EL.D. to the cloud server, where the analysis is performed, is possible via a Bluetooth connection and an Android interface and is sensitive to the substrate employed. A series of blanks, mimicking as good as possible the substrate should be registered prior to any sample analysis. Failing to use the appropriate blank hinders the analytical ability of the method.

### 2.4 Post sample treatment

All filter samples were transferred in sterilized Petri dishes (PALL PD2004705) using alcohol sterilized tweezers inside a laminar flow hood equipped with a high-efficiency particulate air (HEPA) filter. The Petri dish was sealed using parafilm and stored in −20 °C prior to be shipped for analysis at the Cyprus Institute of Neurology and Genetics (CING, Nicosia, Cyprus), where they were stored at −80 °C prior to analysis. In a similar manner, cones from cyclonic samplers were directly sealed and stored in −20 °C prior to being shipped for analysis.

### 2.5 Sampling strategy

The major aim of this study was to assess the positivity levels inside patient wards and ICU treating COVID-19 patients, thus addressing the occupational hazards clinicians faced. As the pandemic progressed, additional wards were included to treat COVID-19 patients, each of which was assessed. However, each new ward had different characteristics with respect to access, personnel and patient cooperation, as well as its spatial configuration and had to be treated individually in order to achieve this study's major goal. For ICU it additionally aimed at investigating AGP, a major clinicians' concern.

A total of 82 air samples were collected at FW using the filter sampler shown in Supp. Fig 1 using Teflon substrates. Sampling was conducted consequently inside the apparel changing room of the clinicians, inside two patient rooms (one 4-bed and one 2-bed) and at the corridor that connects all three areas but just outside of the 4-bed patient room (see Fig. 1). Using the same (filter) sampler in all rooms allowed a direct assessment of positivity rate based on room occupancy; 2-bed vs 4-bed rooms vs no patients at apparel changing room. The sampler located outside the 4-bed patient room was used to determine whether the virus was able to escape from the patient rooms. A dry cyclonic sampler was placed in the middle of the same corridor but opposite (instead of just outside) from patient rooms, to assess whether the virus could be identified even further away from patient rooms.

Environmental samples, as described in Section 2.1, using swabs were collected from frequent used objects including toilet seats, bed rails, litter bin lids, and door handles, while ground (passive) samples were collected using a passive method employing Petri dishes (see also Section 2.1) inside patient and the apparel changing rooms of FW. Swab samples allowed identification of contaminated objects, while passive sampling allowed collection in areas where access to power was not possible such as toilets.

Size segregated air samples were collected using the MOUDI cascade impactor on 8 occasions outside of a 4-bed patient room at FW. Due to noise considerations, the sampler could not be installed inside patient rooms.

Permission to leave unattended devices inside patient rooms at Nicosia's general hospital COVID-19 ward (NW) was not granted due to noise considerations. Instead, samples were drawn for 60 min inside symptomatic adult patient rooms using the Coriolis Micro sampler once per day. The sampler was transferred to the room with the most recent patient addition following the patient's progress till the patient was dismissed from NW and transferred elsewhere. In total, 7 patients were followed for 2–5 days each inside single, double or 4-bed rooms where other patients were also present. The Coriolis Micro sampler was chosen because it operated under high volume (150 Lpm) providing liquid samples that could be directly analyzed at the hospital's PCR facility. The fact that liquid samples were collected also allowed to analysis using B.EL.D. (instead of PCR) within 30 min after sampling. Both methods only reported whether the sample was positive or not without any reference to the viral concentration. In total 25 samples were analyzed using B.EL.D., 14 of which were additionally analyzed by PCR. Additionally, samples of 8-h duration were also taken at the corridor that connected patient rooms at NW using a dry cyclonic sampler and analyzed by RT-PCR, identical to the procedure performed at the corridor of FW, which allowed a direct comparison between these wards.

Filter samples were collected using both Teflon (n = 11) and gelatin (n = 14) substrates at two ICUs at Nicosia General Hospital. Sampling with Teflon substrates lasted 24 h, primarily at NIC1 (8 of the 11), while 1 h sampling using gelatin substrates took place only at NIC2. These samples did not target patients undergoing AGP, and care was taken that at least the 1-h samples did not coincide with any AGP carried out inside the same ICU room. However, it should be assumed that at least one AGP was carried out simultaneously if the sampling period was close to 24 h. Additionally, sampling targeting aerosol-generating procedures was conducted, less than half a meter away from the patient, on 5 occasions inside both ICU's investigated. These patients were selected randomly without any information on their viral load or condition. As a second step, sampling was targeted towards an adult patient in NIC1 that provided an oral swab with sufficient viral load (Ct-value 31), while the remaining patients at NIC1 at that time provided positive samples with lower load (Ct-value 33 or higher). The patient was connected on mechanical ventilation, with endotracheal tube and under sedation. Filter samples using gelatin substrates were collected on two occasions, approximately 30, 80, 160 and 200 cm from that specific patient while AGP was performed.

### 2.6 Ethical considerations

This study was conducted under the approval of the Cyprus national bioethics committee (approval EEBK EΠ 2020.01.123), which allowed us to collect air and environmental samples inside hospitals, provided that the patients' anonymity was secured. During this work, the information collected included: the number of patients inside the cohorts, their sex, and the number of days since their admission, without any personal information disclosed. Apart from the written consent by each ward's head, the patients were notified of their participation to the experiment by at least one poster placed inside their rooms. Their verbal consent was always obtained even though it was not a perquisite of the bioethics approval. In case a complaint was raised, action was taken by removing the sampler from the room completely or by reducing sampling to periods where it would not pose an annoyance (e.g. not during nighttime).

## 3 Results

### 3.1 Samples collected at the Famagusta General Hospital–COVID-19 ward (FW)

Out of the 82 air samples collected at FW using the filter sampler, 28 were positive for SARS-CoV-2 RNA. The summary of those measurements is shown in Table 2. The frequency of positive samples in the 4-bed, 2-bed, apparel changing rooms, and just outside the 4-bed patient room was equal to 50% (n = 24), 29.4% (n = 17), 33.3% (n = 12) and 26.9% (n = 26), respectively. A dry cyclonic sampler was placed in the middle of the same corridor but opposite (instead of just outside) from patient rooms (Fig. 1) and the frequency of positive samples was equal to 33.3% (n = 9).

A summary of the environmental sample results collected at FW is shown in Table 2, while a more detailed account can be found in Table 3. The frequency of positive samples collected using swabs from toilet seats, bed rails, litter bin lids, and door handles was 37.5% (n = 8), 0% (n = 6), 10% (n = 10) and 8.3% (n = 12), respectively. In terms of the ground (passive) samples, SARS-CoV-2 was found in 17% of the samples collected inside the toilet of the 4-bed room at FW (n = 6), 42.8% of the samples collected from the 2-bed patient room (n = 7), 40% of the samples collected at the corridor (n = 5), while none were found in the samples collected from the apparel changing room (n = 3).

**Table 3:** Total and positive environmental samples using swabs and passive samplers at different locations of the Famagusta General Hospital—Ward (FW).

		Inside Patient rooms		Litter bin handles in		Corridor	
		**Bed rails**	**Toilet seat**	**Apparel Room**	**Corridor**	**Floor**	**Handles**
Swabs	Total	6	8	8	2	3	12
	Positive	0	3	1	0	0	1
							
		**4-bed toilet**	**2-bed room**	**Apparel Room**	**Corridor**		
Passive Samples	Total	6	7	3	5		
	Positive	1	3	0	2		

**Fig. 2:**
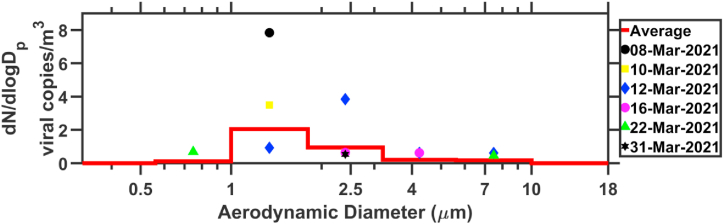
Average size distribution (red line) of COVID-19 positive-only samples collected using a MOUDI. Results from the individual samples are also shown.

Size segregated air samples were collected using a MOUDI cascade impactor on 8 occasions outside of a 4-bed patient room at FW, the overall results of which are summarized in Fig. 2 and shown more extensively in Suppl. Table 1. Virus copies were not identified in two of the samples (25%). In the remaining 6 samples SARS-CoV-2 RNA was primarily identified at sizes between 1.8 and 10 μm. On one sample, viral RNA was identified at sizes 10-18 μm and 1–1.8 μm but not in any size between these two. It is not clear whether the virus was actually present in both modes or the sample was biased due to fractionation, a common discrepancy in sampling with cascade impactors. When particles impact on the substrate surface, occasionally instead of depositing they fractionate into smaller pieces. When biological material is sampled, fractionation may bias the result since the nucleic material or, more frequently, the capsid may shatter, preventing it from being detected (false negative). If a part of the fractionated particle resuspends, it is driven by the flow streamlines to lower impactor stages as if it was a sampled particle of lower size causing a discrepancy.

### 3.2 Samples collected at the Nicosia General Hospital–COVID-19 ward (NW)

At Nicosia's general hospital COVID-19 ward (NW), samples were drawn for 60 min inside symptomatic adult patient rooms using a Coriolis Micro sampler. Positive samples were acquired from only four out of the seven patients followed. The obtained liquid samples were directly sent for analysis at the hospital's PCR facility while a small fraction was analyzed using B.EL.D. within 30 min after sampling. Both methods only reported whether the sample was positive or not without any reference to the viral concentration. In total 25 samples were analyzed using B.EL.D., with approximately 24% (6 out 25) samples found positive. Out of the 25 samples, 14 were additionally analyzed by PCR. There was a general agreement between the two methods (z = 0.41, p = 0.34) based on z-test on proportions [55], with 4 and 9 samples found positive and negative, respectively. Disagreement was identified in one sample detected positive by PCR but negative by B.EL.D. A detailed description of the statistical test can be found in the supplement. Samples of 8-h duration were also taken at the corridor that connects these rooms using a dry cyclonic sampler and analyzed by RT-PCR. Out of the 8 samples acquired, only one was found positive.

### 3.3 Samples collected at two Intensive Care Units

None of the 8 filter samples collected at NIC1 were identified positive. While 3 out of the 17 samples (12%) collected at NIC2, were positive for SARS-CoV-2 RNA (Table 2). These three positive samples were collected on the 14th, 16th and December 17, 2020 in proximity to the same intubated patient each day.

Sampling targeting aerosol-generating procedures was conducted, less than half a meter away from the patient, on 5 occasions inside both ICU's investigated. SARS-CoV-2 RNA was not identified in any of these samples taken from randomly selected adult patients. However, positive samples during AGP were acquired when an adult patient in NIC1 that provided an oral swab with sufficient viral load (Ct-value 31), was targeted. Filter samples using gelatin substrates were collected on two occasions, approximately 30, 80, 160 and 200 cm from that specific patient while AGP was performed. The first set of samples was acquired during endotracheal aspiration and only the sample that was 2 m away was found positive to SARS-CoV-2 RNA. When an ambu bag was utilized, the samples 80 and 160 cm away from the patient were found positive but not the ones 30 and 200 cm away.

## 4 Discussion

This study aimed at healthcare facilities and in CoViD-19 cohorts in specific, where patients were introduced one or more days after admitted to the hospital, until proven SARS-CoV-2 positive. It has been shown that the lowest transmission dynamics are achieved by patients in resting conditions, similar to those hospitalized [56]. Therefore, the results of this study reflect a minimum with respect to viral levels and positivity rates. The fact that all patients were under medical treatment and several intubated, contributed toward this end as well. The major limitation of this study, related to the sampling methods applied, is that no effort to cultivate the virus was made and therefore it could not be determined whether the identified positive samples were a risk to the clinicians.

### 4.1 Airborne SARS-CoV-2 in wards

Inside either of the ICU's investigated, the frequency of positive samples was the lowest observed in this study, in agreement with other studies [[44], [45], [46],^49^]. At NW, the positivity rate inside patient rooms (24%) is higher than that observed in the ICU's (0-12%). Outside the patient rooms at NW, the positivity rate was even lower (12.5%). The highest observed positivity rate in this study was identified at the 4-bed room of FW, which was double compared to the room with half the number of patients (2-bed room) at the same facility. Even though this result was expected, it was not expected that all other locations at FW featured similar positivity rates (27-33%) to the one identified inside 2-bed patient rooms. These locations include the FW's corridor either just outside the 4-bed patient room (where the highest positivity rates were observed), in the middle of that corridor (Fig. 1) and of the apparel changing room. None of these areas was frequently accessed by patients, suggesting that the virus escaped patient rooms. Patient rooms at NW, similar to FW, were equipped with a high flow exhaust system to achieve negative pressure-like conditions. However, at NW the patient room doors were opened only momentarily, unlike at FW where the patient room doors were kept open for relatively prolonged periods. On the contrary, at both ICU's care was taken that patient room doors open as infrequently as possible and for no more than a few seconds, resulting in very low positivity rates. These results suggest that the efficacy of the ventilation system inside patient rooms is hindered if the door is left open for prolonged periods allowing the virus to escape.

This conclusion is further supported by the environmental samples collected at FW. The frequency of positive passive samples is approximately equal to the frequency of positive air samples inside a two-patient room and in the ward's corridor, suggesting a common cause, SARS-CoV-2 being airborne in these areas. In contrast, the positivity of passive samples at the toilet of the 4-bed room was a factor 3 lower than that identified inside that room because, the door that connects the two areas remained largely closed. It should be noted that this toilet was accessed by patients as suggested by the positive samples (using swabs) from the toilet cover. The frequency of positive samples of that toilet seat was the highest compared to other objects used mainly by clinicians including litter bin and door handles. No positive samples were collected from patient bed rails, suggesting that either the specific area sampled was not used by the patients or that disinfection methods applied were effective [57,58].

Passive samples inside the apparel room yielded no positives despite the high frequency (33%) of air samples in the same room. Viral content in apparel changing rooms has been identified early during the pandemic (Liu et al., 2020), suggesting that the clinicians themselves could be potential vectors of the disease. Our results support this finding. However, recent studies failed to identify positive samples [31,47] in such areas.

### 4.2 SARS-CoV-2 size distribution at FW

SARS-CoV-2 RNA was primarily identified at sizes between 1.8 and 10 μm in the majority of the positive size segregated samples. Reports on size-fractionated air samples of SARS-CoV-2, apart from being very few in number, employ samplers with broader size cuts than those presented in this work. The earliest report on size fractionation of SARS-CoV-2 suggested that the virus was primarily located in the submicron range [29]. Inside a car driven by a mildly symptomatic patient, viral RNA was identified in the 0.25–0.50 μm size range [36]. It should be noted that the car windows were closed while air conditioning was employed. A comprehensive nosocomial study by Stern et al. [31] reported that the size ranges at which the virus was detected depended on location. In the ICU and non-CoViD-19 wards of a hospital in Boston, USA, positive samples in sizes above 10 μm were identified. However, in proximity to symptomatic COVID-19 patient rooms the virus was identified between 2.5 and 10 μm only. For a limited (three) number of samples, Chia et al. [30] reported that the viral RNA could be detected only in sizes exceeding 1 μm. Results from both studies agree with those reported in this work. However, in symptomatic patient rooms in Kuwait hospitals, positive samples were identified in sizes above 2.5 μm, exceeding even 10 μm [47], while Santarpia et al. [32] identified positive samples in the entire size spectrum including the submicrometer range. Even though there is no general agreement between studies on the size fraction SARS-CoV-2 lies at, the majority of studies, including this one, report it in a range between 1 and 10 μm.

### 4.3 Towards a rapid detection system of SARS-CoV-2 in air

A total of 14 samples, collected from 31st January till March 5, 2021 at NW, were simultaneously analyzed by both PCR and B.EL.D.. Based on these samples, discussed in Section 3.2, and assuming RT-PCR as the reference method, the sensitivity (the ability of a test to identify correctly positive samples) and specificity (the ability of a test to identify correctly negative samples) of B.EL.D. were equal to 80% (n = 5) and 100% (n = 9), respectively. An assessment of B.EL.D., based on 110 positive and 136 negative samples reported by Apostolou et al. [53], suggested that the B.EL.D. sensitivity and specificity against PCR is 92.7% and 97.8%, respectively), which is not statistically different at 95% CI (p values equal to 0.17 and 0.23, respectively based on z test on proportions) than those reported in this work. These results, which are not conclusive, support that liquid samples from the Coriolis micro sampler, combined with B.EL.D. as the analytical tool may provide a rapid detection method for SARS-CoV-2 in ambient air. This system can act as an early warning system for airborne SARS-CoV-2. However, further investigation is required due to the low population of samples investigated.

### 4.4 Aerosol generating procedures

Only when an adult patient with sufficient viral load (Ct-value 31) was targeted, positive samples related to AGP were acquired. Even though four samples were collected, approximately 30, 80, 160 and 200 cm from that specific patient, during each occasion not all samples were identified positive. It is unclear why samples from all distances were not found positive. Even though, gelatin filters were utilized to reduce sampling discrepancies, these cannot be completely ruled out. In addition, the size distribution of the produced aerosol is unknown and neither the spread of the AGP-generated plume in space during the procedure. The latter is further complicated by the turbulence caused by the ventilation system. These results suggest that endotracheal aspiration and the use of ambu bag are sources of SARS-CoV-2 transmission within a 2 m distance from the patient. Despite the small number of samples collected, these are deemed valuable taking into account that studies aiming at AGP on COVID-19 patients are limited in number. Jerry et al. [50] did not identify positive samples (n = 8) from COVID-19 patients undergoing treatments likely to produce aerosol, while Lei et al. [49] detected one tentative positive sample inside an ICU and in proximity of 1 m to patients undergoing AGP. Both studies did not aim at AGP and neither specified the type of AGP taking place. Tracheostomy has been shown to be a source of viral spread [12], based on a limited number of samples. Similar to this work, the number of samples was limited, and viral RNA was not identified on all samples collected during the AGP. The major difference between the two studies is the detected levels of viral RNA. The measured concentrations in each of the positive samples reported in this study were relatively low, ranging from 44 to 67 copies m^−3^. This amount is 17-fold lower than that reported by Zhou et al. [12] but for different AGP than that targeted in this work.

The fact that SARS-CoV-2 RNA was not identified in all of the samples distributed from the patient undergoing AGP, and that the viral levels were low in the positive samples, can also be explained by the presence of eddies due to a robust ventilation system. It also suggests that the 5 negative samples collected during other AGP procedures may have been simply positioned at locations/distances from the patient that did not allow identification of the virus even though viral spread was occurring. The inhomogeneity of viral spread should be taken into account in future studies concerning AGP. Considering that the frequency of positive samples observed in either ICU investigated was low (0-18%) despite applying AGP's on a daily basis, supports the argument that the ventilation system applied is an effective mitigation method even if sources of viral spread are present. It should be noted that ventilation system in either ICU enforced 16 regenerations per hour, instead of 14 at the wards, and that foot traffic inside patient rooms was limited to the minimum.

### 4.5 Viral levels within wards and ICUs

**Fig. 3:**
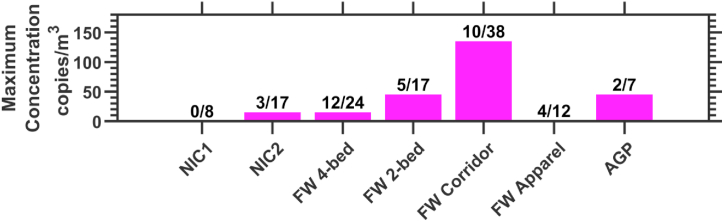
Maximum concentrations measured in each of the locations inside the clinics investigated. The number of positive/total number of samples in each location is also shown.

The highest concentration, equal to 135 copies m^−3^, was identified at the corridor of FW, outside a 4-bed patient room, which is lower than the 2000 copies m^−3^ reported by Chia et al. [30], but higher than values reported in most other studies. A summary of the maximum observed levels in each of the locations monitored can be found in Fig. 3. Inside a two-bed patient cohort at FW, the highest observed concentration was approximately equal to 45 copies m^−3^, similar to that identified during AGP's and that reported by Liu et al. [29] (42 copies m^−3^) and Stern et al. [31] (51 copies m^−3^). At both, the intensive care unit (NIC2) and the FW's 4-bed cohort, the highest concentration observed was 15 copies m^−3^, even though at the latter the highest and at the former the lowest positivity rates were observed in this study, respectively. Inside an ICU in Kuwait, Stern et al. [31] reported a maximum concentration equal to 25 copies m^−3^.

Most of the positive samples in this study were at the limit of quantitation of the analytical method described in the supplement which corresponds to less than 8 viral copies per sample, with only 4 of the positive samples exceeding this threshold. As a result, the concentration in air was largely dictated by the sampling duration. In the apparel changing room of FW, where the sampling typically lasted for 48 h, the highest observed concentration was less than 1 copy m^−3^. Taking all of the above into account, the concentrations listed in Fig. 3, should also be regarded as infrequent.

The infectious dose of SARS-CoV-2 is yet to be determined but it is possibly similar to that of SARS-CoV-1 [59]. In this case, 50% of the population exposed to 280 viral particles should be infected [60]. Only one air sample of the 165 collected, identified outside a 4-bed patient cohort at FW, exceeded this threshold. Even though technical limitations decreasing the viral load detection, including extraction efficiency, PCR sensitivity, as well as RNA degradation during sampling cannot be ruled out, they cannot account for the deficit to reach the 280 viral limit. An overall detection efficiency of 3% or less would be required.

The low concentrations reported in this study are partly the result of a robust ventilations system, 16 and 14 regenerations using full fresh air per hour at intensive care units and CoViD-19 wards, respectively, which diluted the viral load. The likely episodic nature of SARS-CoV-2 transmission [31] could be another cause. Under these conditions, the virus was identified in supermicron sizes only (Section 4.2) at least 5 m from the source. It is common for particles of this size to travel beyond 2 m [61]. This could be enhanced by the prolonged periods the patient room door remained opened due to intense foot traffic in that ward.

## 5 Conclusions

The positivity rate of wards with adult COVID-19 patients ranged from 50% inside a 4-bed cohort and 24-33% elsewhere (Section 4.1). At two ICUs the positivity was even lower reaching a maximum of 18%. The vast majority of positive samples were at the detection limit of the method applied to identify SARS-CoV-2 in ambient air (Section 4.5). The observed low viral concentrations are partly the result of the robust ventilation system applied in all facilities monitored in this study. In one ward due to increased foot traffic, the door of a 4-bed cohort was left open for prolonged periods. The positivity rate just outside this cohort was similar to that inside 2-bed cohort of the same ward (Section 4.1), suggesting that the virus is able to escape despite the robust ventilation system designed to keep patient rooms under negative pressure. In the same area, just outside the 4-bed cohort, the highest viral concentration of SARS-CoV-2 was detected suggesting that hindering the ventilation system puts healthcare workers at risk. In a different ward applying similar precaution measures, but with less foot traffic, the frequency of positive samples in the ward's corridor was almost half. It should be noted that SARS-CoV-2 was found to lie predominantly in sizes between 1 and 2.5 μm in diameter, which are adequately diffusive to travel distances longer than a few meters (Section 4.2).

AGP were found to be a source of viral spread only when targeted towards a patient with sufficient viral load (Ct-value 31). Two types of AGP were carried out, endotracheal aspiration and the use of ambu bag, during which positive samples within a 2 m distance from the patient were identified (Section 4.4). However, out of the 4 samples distributed across the 2 m distance, not all were found positive and those positive were close to the detection limit of the applied method. These results suggest that at least the two types of AGPs investigated are a source of viral spread but the ventilation system applied diluted the sample and created eddies that distribute the load non-uniformly. Inside ICU's foot traffic is reduced to the minimum and the patient room door is opened only momentarily, and as a result the frequency of positive samples was low despite applying AGP on a daily basis.

Finally, this study has successfully managed to identify SARS-CoV-2 RNA in air within less than 1 h after sampling by utilizing a combination of commercial instruments; a Coriolis micro air sampler and B.EL.D., a cell-based biosensor (Section 4.3). Even though this method does not provide information on the viral levels, it can be used as an early warning system for airborne SARS-CoV-2 RNA.

## Author contribution statement

Rafail Konatzii: Performed the experiments; Wrote the paper.

Fabian Schmidt-Ott; Panagiotis Stagianos: Performed the experiments.

Lakis Palazis; Maria Foka: Conceived and designed the experiments.

Jan Richter; Christina Christodoulou: Contributed reagents, materials, analysis tools or data.

Jean Sciare: Conceived and designed the experiments; Contributed reagents, materials, analysis tools or data.

Michael Pikridas: Conceived and designed the experiments; Analyzed and interpreted the data; Wrote the paper.

## Funding statement

Jean Sciare and Michael Pikridas were supported by AIR-COVID NETWORK (CONCEPT-COVID/0420/0014) and CURE-SARS (CONCEPT COVID/0420/0015) projects, which are co-financed by the European Regional Development Fund and the Republic of Cyprus through the Research and Innovation Foundation.

This study has also been supported financially by the H2020-EMME-CARE (GA 856612).

## Data availability statement

Data are available at https://doi.org/10.5281/zenodo.7551776.

## Declaration of interest's statement

The authors declare the following conflict of interests: Lakis Palazis and Maria Foka work at the ICU of Nicosia General Hospital. All other authors declare no competing interests.

## References

[1] World Health Organization (2003). https://www.who.int/news/item/12-03-2003-who-issues-a-global-alert-about-cases-of-atypical-pneumonia.

[2] Huff H.V., Singh A. (2020). Asymptomatic transmission during the Coronavirus disease 2019 pandemic and implications for public health strategies. Clin. Infect. Dis..

[3] Zhao H.J., Lu X.X., Deng Y. Bin, Tang Y.J., Lu J.C. (2020). COVID-19: asymptomatic carrier transmission is an underestimated problem. Epidemiol. Infect..

[4] (2022). https://www.cdc.gov/infectioncontrol/guidelines/environmental/appendix/air.html#tableb1.

[5] Leung N.H.L., Chu D.K.W., Shiu E.Y.C., Chan K.-H., McDevitt J.J., Hau B.J.P., Yen H.-L., Li Y., Ip D.K.M., Peiris J.S.M., Seto W.-H., Leung G.M., Milton D.K., Cowling B.J. (2020). Respiratory virus shedding in exhaled breath and efficacy of face masks. Nat. Med..

[6] Wong S.C.Y., Kwong R.T.S., Wu T.C., Chan J.W.M., Chu M.Y., Lee S.Y., Wong H.Y., Lung D.C. (2020). Risk of nosocomial transmission of coronavirus disease 2019: an experience in a general ward setting in Hong Kong. J. Hosp. Infect..

[7] Wang X., Zhou Q., He Y., Liu L., Ma X., Wei X., Jiang Nanchuan, Liang L., Zheng Y., Ma L., Xu Y., Yang D., Zhang J., Yang B., Jiang Ning, Deng T., Zhai B., Gao Y., Liu W., Bai X., Pan T., Wang G., Chang Y., Chang Y., Zhang Z., Zhang Z., Shi H., Ma W.L., Gao Z. (2020). Nosocomial outbreak of COVID-19 pneumonia in Wuhan, China. Eur. Respir. J..

[8] Arons M.M., Hatfield K.M., Reddy S.C., Kimball A., James A., Jacobs J.R., Taylor J., Spicer K., Bardossy A.C., Oakley L.P., Tanwar S., Dyal J.W., Harney J., Chisty Z., Bell J.M., Methner M., Paul P., Carlson C.M., McLaughlin H.P., Thornburg N., Tong S., Tamin A., Tao Y., Uehara A., Harcourt J., Clark S., Brostrom-Smith C., Page L.C., Kay M., Lewis J., Montgomery P., Stone N.D., Clark T.A., Honein M.A., Duchin J.S., Jernigan J.A. (2020). Presymptomatic SARS-CoV-2 infections and transmission in a skilled nursing facility. N. Engl. J. Med..

[9] Meredith L.W., Hamilton W.L., Warne B., Houldcroft C.J., Hosmillo M., Jahun A.S., Curran M.D., Parmar S., Caller L.G., Caddy S.L., Khokhar F.A., Yakovleva A., Hall G., Feltwell T., Forrest S., Sridhar S., Weekes M.P., Baker S., Brown N., Moore E., Popay A., Roddick I., Reacher M., Gouliouris T., Peacock S.J., Dougan G., Torok M.E., Goodfellow I. (2020). Rapid implementation of SARS-CoV-2 sequencing to investigate cases of health-care associated COVID-19: a prospective genomic surveillance study. Lancet Infect. Dis..

[10] Van Praet J.T., Claeys B., Coene A.S., Floré K., Reynders M. (2020). Prevention of nosocomial COVID-19: another challenge of the pandemic. Infect. Control Hosp. Epidemiol..

[11] Wang D., Hu B., Hu C., Zhu F., Liu X., Zhang J., Wang B., Xiang H., Cheng Z., Xiong Y., Zhao Y., Li Y., Wang X., Peng Z. (2020). Clinical characteristics of 138 hospitalized patients with 2019 novel coronavirus–infected pneumonia in Wuhan, China. JAMA.

[12] Zhou F., Yu T., Du R., Fan G., Liu Y., Liu Z., Xiang J., Wang Y., Song B., Gu X., Guan L., Wei Y., Li H., Wu X., Xu J., Tu S., Zhang Y., Chen H., Cao B. (2020). Clinical course and risk factors for mortality of adult inpatients with COVID-19 in Wuhan, China: a retrospective cohort study. Lancet.

[13] Lumley S.F., Constantinides B., Sanderson N., Rodger G., Street T.L., Swann J., Chau K.K., Odonnell D., Warren F., Hoosdally S., Odonnell A.M., Walker T.M., Stoesser N.E., Butcher L., Peto T.E., Crook D.W., Jeffery K., Matthews P.C., Eyre D.W. (2021). Epidemiological data and genome sequencing reveals that nosocomial transmission of SARS-CoV-2 is underestimated and mostly mediated by a small number of highly infectious individuals. J. Infect..

[14] Maleki M., Anvari E., Hopke P.K., Noorimotlagh Z., Mirzaee S.A. (2021). An updated systematic review on the association between atmospheric particulate matter pollution and prevalence of SARS-CoV-2. Environ. Res..

[15] China CDC Weekly (2020). http://weekly.chinacdc.cn/en/article/id/e53946e2-c6c4-41e9-9a9b-fea8db1a8f51.

[16] Guan W., Ni Z., Hu Yu, Liang W., Ou C., He J., Liu L., Shan H., Lei C., Hui D.S.C., Du B., Li L., Zeng G., Yuen K.-Y., Chen R., Tang C., Wang T., Chen P., Xiang J., Li S., Wang Jin-lin, Liang Z., Peng Y., Wei L., Liu Y., Hu Ya-hua, Peng P., Wang Jian-ming, Liu J., Chen Z., Li G., Zheng Z., Qiu S., Luo J., Ye C., Zhu S., Zhong N. (2020). Clinical characteristics of coronavirus disease 2019 in China. N. Engl. J. Med..

[17] Eyre D.W., Lumley S.F., Odonnell D., Campbell M., Sims E., Lawson E., Warren F., James T., Cox S., Howarth A., Doherty G., Hatch S.B., Kavanagh J., Chau K.K., Fowler P.W., Swann J., Volk D., Yang-Turner F., Stoesser N.E., Matthews P.C., Dudareva M., Davies T., Shaw R.H., Peto L., Downs L.O., Vogt A., Amini A., Young B.C., Drennan P., Mentzer A.J., Skelly D., Karpe F., Neville M.J., Andersson M., Brent A.J., Jones N., Ferreira L.M., Christott T., Marsden B.D., Hoosdally S., Cornall R., Crook D.W., Stuart D.I., Screaton G., Peto T.E.A., Holthof B., O’donnell A.M., Ebner D., Conlon C.P., Jeffery K., Walker T.M., Watson A.J.R., Taylor A., Chetwynd A., Grassam-Rowe A., Mighiu A.S., Livingstone A., Killen A., Rigler C., Harries C., East C., Lee C., Mason C.J.B., Holland C., Thompson C., Hennesey C., Savva C., Kim D.S., Harris E.W.A., McGivern E.J., Qian E., Rothwell E., Back F., Kelly G., Watson G., Howgego G., Chase H., Danbury H., Laurenson-Schafer H., Ward H.L., Hendron H., Vorley I.C., Tol I., Gunnell J., Ward J.L.F., Drake J., Wilson J.D., Morton J., Dequaire J., O'Byrne K., Motohashi K., Harper K., Ravi K., Millar L.J., Peck L.J., Oliver M., English M.R., Kumarendran M., Wedlich M., Ambler O., Deal O.T., Sweeney O., Cowie P., te Water Naudé R., Young R., Freer R., Scott S., Sussmes S., Peters S., Pattenden S., Waite S., Johnson S.A., Kourdov S., Santos-Paulo S., Dimitrov S., Kerneis S., Ahmed-Firani T., King T.B., Ritter T.G., Foord T.H., De Toledo Z., Christie T. (2020). Differential occupational risks to healthcare workers from SARS-CoV-2 observed during a prospective observational study. Elife.

[18] Shah A.S.V., Wood R., Gribben C., Caldwell D., Bishop J., Weir A., Kennedy S., Reid M., Smith-Palmer A., Goldberg D., McMenamin J., Fischbacher C., Robertson C., Hutchinson S., McKeigue P., Colhoun H., McAllister D.A. (2020). Risk of hospital admission with coronavirus disease 2019 in healthcare workers and their households: nationwide linkage cohort study. BMJ.

[19] Wang C.C., Prather K.A., Sznitman J., Jimenez J.L., Lakdawala S.S., Tufekci Z., Marr L.C. (2021). Airborne transmission of respiratory viruses. Science.

[20] Wells W.F. (1934). ON air-borne infection: study II. Droplets and droplet nuclei. Am. J. Epidemiol..

[21] Seinfeld J.H., Pandis S.N. (2016).

[22] Santarpia A.J.L., Rivera D.N., Herrera V., Morwitzer M.J., Creager H., Santarpia G.W., Crown K.K., Brett-major D.M., Broadhurst M.J., Lawler J.V., Reid S.P., Lowe J.J. (2020).

[23] Morawska L., Cao J. (2020). Airborne transmission of SARS-CoV-2: the world should face the reality. Environ. Int..

[24] Morawska L., Milton D.K. (2020). It is time to address airborne transmission of coronavirus disease 2019 (COVID-19). Clin. Infect. Dis..

[25] Noorimotlagh Z., Jaafarzadeh N., Martinez S.S., Mirzaee S.A. (2021). A systematic review of possible airborne transmission of the COVID-19 virus (SARS-CoV-2) in the indoor air environment. Environ. Res..

[26] Stadnytskyi V., Bax C.E., Bax A., Anfinrud P. (2020). The airborne lifetime of small speech droplets and their potential importance in SARS-CoV-2 transmission. Proc. Natl. Acad. Sci. U.S.A..

[27] Bahl P., de Silva C., Bhattacharjee S., Stone H., Doolan C., Chughtai A.A., MacIntyre C.R. (2021). Droplets and aerosols generated by singing and the risk of coronavirus disease 2019 for choirs. Clin. Infect. Dis..

[28] Miller S.L., Nazaroff W.W., Jimenez J.L., Boerstra A., Buonanno G., Dancer S.J., Kurnitski J., Marr L.C., Morawska L., Noakes C. (2021). Transmission of SARS-CoV-2 by inhalation of respiratory aerosol in the Skagit Valley Chorale superspreading event. Indoor Air.

[29] Liu Yuan, Ning Z., Chen Y., Guo M., Liu Yingle, Gali N.K., Sun L., Duan Y., Cai J., Westerdahl D., Liu X., Xu K., Ho K.fai, Kan H., Fu Q., Lan K. (2020). Aerodynamic analysis of SARS-CoV-2 in two Wuhan hospitals. Nature.

[30] Chia P.Y., Coleman K.K., Tan Y.K., Ong S.W.X., Gum M., Lau S.K., Lim X.F., Lim A.S., Sutjipto S., Lee P.H., Son T.T., Young B.E., Milton D.K., Gray G.C., Schuster S., Barkham T., De P.P., Vasoo S., Chan M., Ang B.S.P., Tan B.H., Leo Y.S., Ng O.T., Wong M.S.Y., Marimuthu K., Lye D.C., Lim P.L., Lee C.C., Ling L.M., Lee L., Lee T.H., Wong C.S., Sadarangani S., Lin R.J., Ng D.H.L., Sadasiv M., Yeo T.W., Choy C.Y., Tan G.S.E., Dimatatac F., Santos I.F., Go C.J., Chan Y.K., Tay J.Y., Tan J.Y.L., Pandit N., Ho B.C.H., Mendis S., Chen Y.Y.C., Abdad M.Y., Moses D. (2020). Detection of air and surface contamination by SARS-CoV-2 in hospital rooms of infected patients. Nat. Commun..

[31] Stern R.A., Al-Hemoud A., Alahmad B., Koutrakis P. (2021). Levels and particle size distribution of airborne SARS-CoV-2 at a healthcare facility in Kuwait. Sci. Total Environ..

[32] Santarpia J.L., Herrera V.L., Rivera D.N., Ratnesar-shumate S., Reid P., Denton P.W., Martens J.W.S., Fang Y., Conoan N., Callahan V., Lawler J.V., Brett-major D.M., Lowe J.J. (2020).

[33] Ding Z., Qian H., Xu B., Huang Y., Miao T., Yen H.L., Xiao S., Cui L., Wu X., Shao W., Song Y., Sha L., Zhou L., Xu Y., Zhu B., Li Y. (2020). Toilets dominate environmental detection of SARS-CoV-2 virus in a hospital. medRxiv.

[34] Kenarkoohi A., Noorimotlagh Z., Falahi S., Amarloei A., Mirzaee S.A., Pakzad I., Bastani E. (2020). Hospital indoor air quality monitoring for the detection of SARS-CoV-2 (COVID-19) virus. Sci. Total Environ..

[35] Binder R.A., Alarja N.A., Robie E.R., Kochek K.E., Xiu L., Rocha-Melogno L., Abdelgadir A., Goli S.V., Farrell A.S., Coleman K.K., Turner A.L., Lautredou C.C., Lednicky J.A., Lee M.J., Polage C.R., Simmons R.A., Deshusses M.A., Anderson B.D., Gray G.C. (2020). Environmental and aerosolized severe acute respiratory syndrome coronavirus 2 among hospitalized coronavirus disease 2019 patients. J. Infect. Dis..

[36] Lednicky J.A., Lauzardo M., Alam M.M., Elbadry M.A., Stephenson C.J., Gibson J.C., Morris J.G. (2021). Isolation of SARS-CoV-2 from the air in a car driven by a COVID patient with mild illness. Int. J. Infect. Dis..

[37] Razzini K., Castrica M., Menchetti L., Maggi L., Negroni L., Orfeo N.V., Pizzoccheri A., Stocco M., Muttini S., Balzaretti C.M. (2020). SARS-CoV-2 RNA detection in the air and on surfaces in the COVID-19 ward of a hospital in Milan. Italy. Sci. Total Environ..

[38] Li Y.H., Fan Y.Z., Jiang L., Wang H.B. (2020). Aerosol and environmental surface monitoring for SARS-CoV-2 RNA in a designated hospital for severe COVID-19 patients. Epidemiol. Infect..

[39] Ong S.W.X., Tan Y.K., Chia P.Y., Lee T.H., Ng O.T., Wong M.S.Y., Marimuthu K. (2020). Air, surface environmental, and personal protective equipment contamination by severe acute respiratory syndrome coronavirus 2 (SARS-CoV-2) from a symptomatic patient. JAMA.

[40] Cheng V.C.-C., Wong S.-C., Chan V.W.-M., So S.Y.-C., Chen J.H.-K., Yip C.C.-Y., Chan K.-H., Chu H., Chung T.W.-H., Sridhar S., To K.K.-W., Chan J.F.-W., Hung I.F.-N., Ho P.-L., Yuen K.-Y. (2020). Air and environmental sampling for SARS-CoV-2 around hospitalized patients with coronavirus disease 2019 (COVID-19). Infect. Control Hosp. Epidemiol..

[41] Faridi S., Niazi S., Sadeghi K., Naddafi K., Yavarian J., Shamsipour M., Jandaghi N.Z.S., Sadeghniiat K., Nabizadeh R., Yunesian M., Momeniha F., Mokamel A., Hassanvand M.S., MokhtariAzad T. (2020). A field indoor air measurement of SARS-CoV-2 in the patient rooms of the largest hospital in Iran. Sci. Total Environ..

[42] Masoumbeigi H., Ghanizadeh G., Yousefi Arfaei R., Heydari S., Goodarzi H., Dorostkar Sari R., Tat M. (2020). Investigation of hospital indoor air quality for the presence of SARS-Cov-2. J. Environ. Heal. Sci. Eng..

[43] Vosoughi M., Karami C., Dargahi A., Jeddi F., Jalali K.M., Hadisi A., Haghighi S.B., Dogahe H.P., Noorimotlagh Z., Mirzaee S.A. (2021). Investigation of SARS-CoV-2 in hospital indoor air of COVID-19 patients' ward with impinger method. Environ. Sci. Pollut. Res..

[44] Siegel J.D., Rhinehart E., Jackson M., Chiarello L. (2007). 2007 guideline for isolation precautions: preventing transmission of infectious agents in health care settings. Am. J. Infect. Control.

[45] Smith P.W., Anderson A.O., Christopher G.W., Cieslak T.J., Devreede G.J., Fosdick G.A., Greiner C.B., Hauser J.M., Hinrichs S.H., Huebner K.D., Iwen P.C., Jourdan D.R., Kortepeter M.G., Landon V.P., Lenaghan P.A., Leopold R.E., Marklund L.A., Martin J.W., Medcalf S.J., Mussack R.J., Neal R.H., Ribner B.S., Richmond J.Y., Rogge C., Daly L.A., Roselle G.A., Rupp M.E., Sambol A.R., Schaefer J.E., Sibley J., Streifel A.J., Essen S.G. Von, Warfield K.L. (2006). Designing a biocontainment unit to care for patients with serious communicable diseases: a consensus statement. Biosecur. Bioterrorism Biodefense Strategy, Pract. Sci..

[46] Lee J.K., Jeong H.W. (2020). Rapid expansion of temporary, reliable airborne-infection isolation rooms with negative air machines for critical COVID-19 patients. Am. J. Infect. Control.

[47] Stern R.A., Koutrakis P., Martins M.A.G., Lemos B., Dowd S.E., Sunderland E.M., Garshick E. (2021). Characterization of hospital airborne SARS-CoV-2. Respir. Res..

[48] Klompas M., Baker M.A., Rhee C. (2020). Airborne transmission of SARS-CoV-2. JAMA.

[49] Lei H., Ye F., Liu X., Huang Z., Ling S., Jiang Z., Cheng J., Huang X., Wu Q., Wu S., Xie Y., Xiao C., Ye D., Yang Z., Li Y., Leung N.H.L., Cowling B.J., He J., Wong S.S., Zanin M. (2020). SARS-CoV-2 environmental contamination associated with persistently infected COVID-19 patients. Influenza Other Respi. Viruses.

[50] Jerry J., O'Regan E., O'Sullivan L., Lynch M., Brady D. (2020). Do established infection prevention and control measures prevent spread of SARS-CoV-2 to the hospital environment beyond the patient room?. J. Hosp. Infect..

[51] Marple V.A., Rubow K.L., Behm S.M. (1991). A microorifice uniform deposit impactor (moudi): description, calibration, and use. Aerosol Sci. Technol..

[52] He X., Lau E.H.Y., Wu P., Deng X., Wang J., Hao X., Lau Y.C., Wong J.Y., Guan Y., Tan X., Mo X., Chen Y., Liao B., Chen W., Hu F., Zhang Q., Zhong M., Wu Y., Zhao L., Zhang F., Cowling B.J., Li F., Leung G.M. (2020). Temporal dynamics in viral shedding and transmissibility of COVID-19. Nat. Med..

[53] Apostolou T., Kyritsi M., Vontas A., Loizou K., Hadjilouka A., Speletas M., Mouchtouri V., Hadjichristodoulou C. (2021). Development and performance characteristics evaluation of a new Bioelectric Recognition Assay (BERA) method for rapid Sars-CoV-2 detection in clinical samples. J. Virol. Methods.

[54] Kintzios S., Pistola E., Panagiotopoulos P., Bomsel M., Alexandropoulos N., Bem F., Ekonomou G., Biselis J., Levin R. (2001). Bioelectric recognition assay (BERA). Biosens. Bioelectron..

[55] Montgomery D.C., Runger G.C. (2014).

[56] Buonanno G., Stabile L., Morawska L. (2020). Estimation of airborne viral emission: quanta emission rate of SARS-CoV-2 for infection risk assessment. Environ. Int..

[57] Noorimotlagh Z., Mirzaee S.A., Jaafarzadeh N., Maleki M., Kalvandi G., Karami C. (2021). A systematic review of emerging human coronavirus (SARS-CoV-2) outbreak: focus on disinfection methods, environmental survival, and control and prevention strategies. Environ. Sci. Pollut. Res..

[58] Seif Faezeh, Noorimotlagh Z., Mirzaee S.A., Kalantar M., Barati B., Fard M.E., Fard N.K. (2021). The SARS-CoV-2 (COVID-19) pandemic in hospital: an insight into environmental surfaces contamination, disinfectants' efficiency, and estimation of plastic waste production. Environ. Res..

[59] Beggs C.B. (2020). Is there an airborne component to the transmission of COVID-19?: a quantitative analysis study. medRxiv.

[60] Watanabe T., Bartrand T.A., Weir M.H., Omura T., Haas C.N. (2010). Development of a dose-response Model for SARS coronavirus. Risk Anal..

[61] Tang J.W., Bahnfleth W.P., Bluyssen P.M., Buonanno G., Jimenez J.L., Kurnitski J., Li Y., Miller S., Sekhar C., Morawska L., Marr L.C., Melikov A.K., Nazaroff W.W., Nielsen P.V., Tellier R., Wargocki P., Dancer S.J. (2021). Dismantling myths on the airborne transmission of severe acute respiratory syndrome coronavirus-2 (SARS-CoV-2). J. Hosp. Infect..

