# Supplementary material for: Exposure to airborne SARS-CoV-2 in four hospital wards and ICUs of Cyprus. A detailed study accounting for day-to-day operations and aerosol generating procedures
Source: Heliyon. 2023 Feb 11;9(3):e13669. doi: 10.1016/j.heliyon.2023.e13669 (PMC9918438; doi:10.1016/j.heliyon.2023.e13669)
Supplement: Supplementary file 1 [file mmc1.pdf]

Supplemental information regarding "Exposure to airborne SARS-CoV-2 in four hospital wards and ICUs of Cyprus. A detailed study accounting for day-to-day operations and aerosol generating procedures" by

Rafail Konatzii[1], Fabian Schmidt-Ott[1,4], Lakis Palazis[2], Panagiotis Stagianos[1], Maria Foka[2], Jan Richter[3], Christina Christodoulou[3], Jean Sciare[1] and Michael Pikridas[1]

[1]Climate and Atmosphere Research Centre, The Cyprus Institute, Nicosia, 2121, Cyprus
[2] Department of Intensive Care Unit, Nicosia General Hospital, Ministry of Health, Nicosia, 2029, Cyprus
[3] Department of Molecular Virology, The Cyprus Institute of Neurology and Genetics, Nicosia, 1683, Cyprus
[4] Department of Physics, University of Helsinki, Helsinki, 00014, Finland

1. Comparison of dry cyclonic and filter samplers

Dry cyclonic samples were simultaneously collected with filter ones inside the 4 patient room only on 8 occasions, during which sample duration was limited to 8 hours, allowing for direct comparison between the two samplers. Only gelatin filters were used for this test. The 4 patient room was selected because it exhibited the highest frequency of positive samples of all the areas monitored. On 5 occasions, both methods agreed, 3 of which concerned positive samples. On two occasions, SARS-CoV-2 RNA was identified on the filter sample but not on the ones collected by the cyclonic sampler, despite the higher flowrate by a 10fold of the latter. On one occasion, viral content was identified on the cyclonic sample but not on the filter one.

Discrepancies between different types of samplers are known (Verreault et al., 2008), therefore the difference in these results is not surprising. To the best of our knowledge, there is no reference method for collecting viral content from ambient air. The aforementioned discrepancy can be explained based on differences regarding extraction efficiency from the different substrates.

It should be noted that the absolute viral content (without accounting for the sampled flow) in these samples was equal, even though the dry cyclonic sampler employs a 10 fold higher flowrate that the filter samples. One tentative explanation is that the virus RNA degrades during sampling, preventing identification, and only viruses collected during the end of the sampling period are being detected. This explanation assumes that viruses are accumulated on the samples at the presence of a continuous source. An alternative explanation assumes that patients emit SARS-CoV-2 episodically (e.g. when they cough) and thus the concentration of the sample depends more on the number and intensity of those episodes. An episodic spread of the virus in low concentrations (due to the exhaust system enforced) may also explain the observed discrepancy between the filter and cyclonic samplers.

This comparison was beyond the goal of this study and aimed at pinpointing differences between the two methods. Hence, only a small number of simultaneous samples was collected. The main conclusions is that both sampling methods involve weaknesses that have not been identified that result in false negative identifications.

2. Analytical Methods

RNA extraction and reverse transcription quantitative polymerase chain reaction (RT-qPCR) were performed at the Cyprus Institute of Neurology and Genetics (CING, Nicosia, Cyprus) following the CDC assay targeting the N-gene. In brief, viral RNA was extracted using the Zymo Research Quick-DNA/RNA Viral MagBead Kit. For this purpose, the filter was inserted into a 2ml screw cap Eppendorf tube to which 400 µl 1X DNA/RNA Shield and 4 µl Proteinase K were added to the sample as per manufacturer's instructions and placed on a vortex shaker at room temperature. After 15 min, 800 µl Viral DNA/RNA buffer were then added to each tube and again placed on a vortex shaker for another 10 min. The filter was then removed from the tube and placed in a new clean tube and centrifuged to collect any remaining liquid, which was added to the original tube. 20 µl MagBinding Beads were then added and the samples were loaded for further processing on the Kingfisher Flex purification instrument. RNA was eluted in a final volume of 50 µl.

For detection of SARS-CoV-2 RNA and the parallel detection of human DNA, an in-house duplex Real-Time RT-PCR assay was employed. The primers/probe sets utilized are based on the highly sensitive CDC novel Coronavirus RT-PCR panel that targets a conserved segment of the viral nucleocapsid gene (N1) as well as the human RNase P gene. In short, 20 µl of extracted RNA/DNA were used in a one-step RT-PCR reaction containing 25 µl 2x AgPath-ID Mastermix (ThermoFisher, USA), 2 µl Enzyme mix, 0.4 µM of forward and reverse primers and 0.15 µM of probes. Reverse transcription was carried out on a Quantstudio 5 Real-Time PCR System (ThermoFisher, USA) at 50°C for 15 min followed by 45 cycles of 95 °C for 10 s denaturation plus 60 °C for 30s annealing/extension. For quantification, a standard curve was created using 10-fold serial dilution series of a quantified SARS-CoV2 reference strain in each run.

Liquid samples obtained by the wet cyclonic sampler, were analyzed at the Nicosia General Hospital following a simpler protocol since no extraction was necessary. The VIASURE SARS-CoV-2 Real-Time PCR Detection Kit, which was designed for the specific identification and differentiation of SARS-CoV-2 in respiratory samples from patients with signs and symptoms of COVID-19 infection, was used. Detection was performed in one-step real-time format where the reverse transcription and the subsequent amplification of specific target sequence occurred in the same reaction well. The isolated RNA target was transcribed generating complementary DNA by reverse transcriptase, followed by the amplification of a conserved region of ORF1ab and N genes for SARS-CoV-2 using specific primers and a fluorescent-labelled probe. The test has a detection limit of ≥10 RNA copies per reaction for ORF1ab and N genes. The test has a detection limit of ≥10 RNA copies per reaction for ORF1ab and N genes. The Rotor-Gene Q MDx Real-time PCR cycler with 6 channels was used for these samples.

3. Statistics

The majority of positive samples was at the limit of detection of the PCR method applied, or the method applied inherently was able to provide only qualitative results (see e.g. Section 3.4). Therefore, the results in this study were treated as Boolean (positive, negative). In order to perform comparisons between methods and samplers, a z test on proportions (Montgomery and Runger, 2014) was applied at the 95% confidence interval based on Eq. 1

$$Z = \frac{(p_1 - p_2) - 0}{\sqrt{p_t(1-p_t)(\frac{1}{n_1} + \frac{1}{n_2})}} \quad \text{Eq. 1}$$

In this case, the hypothesized difference is 0 and is compared against the sample difference (p_1-p_2), where p represents the proportion of success for groups 1 and 2. The subscript t refers to pooled proportion of success for both samples. n_1 and n_2 refer to the population of the each of the two groups under investigation. The null hypothesis (H_0) for the test is that the proportions are the same.

Supplemental Table 1. Results from the 8 size fractionated samples collected outside a 4-bed non intubated patient room. The number in color refer to the viral RNA copies identified in each sample. The sampling volume is also shown to deduct the overall concentration in air.

Size Bin (μm)	8th March	8th March	10th March	12th March	16th March	22nd March	26th March	31st March	Overall Positivity
>18	0	0	0	0	0	0	0	0	0%
10.0-18.0	0	0	0	0	0	**7.5**	0	0	12.5%
5.6-10.0	**15**	0	**27.5**	**7.5**	0	0	0	0	37.5%
3.2-5.6	0	0	**<7.5**	**30**	**<7.5**	0	0	**<7.5**	37.5%
1.8-3.2	0	0	0	**<7.5**	**<7.5**	0	0	0	25.0%
1-1.8	0	0	0	**<7.5**	0	**<7.5**	0	0	25.0%
0.56-1.0	0	0	0	0	0	0	0	0	0%
0.32-0.56	0	0	0	0	0	0	0	0	0%
**Total Volume (m^3)**	3.3	8.5	13.5	13.9	13.9	18.7	21.8	16.7	

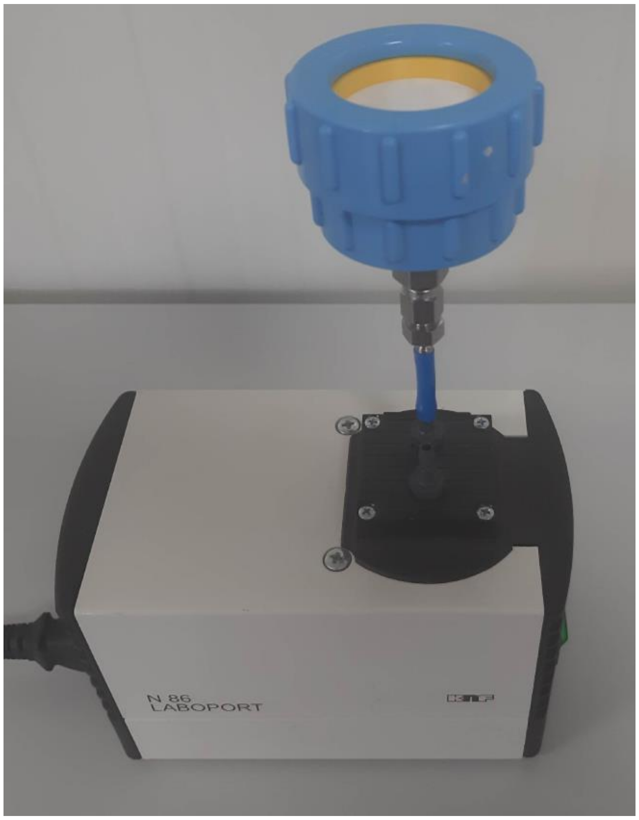

Supplemental Figure 1. Custom made filter sampler employed in this study. The sampler was modified to be used as an open face filter to reduce particle velocity without compromising flow.
